# The Centre of Christmas Blessedness

**Published:** 1913-12-27

**Authors:** 


### The Hospital

The Workers' Newspaper of Administrative Medicine and Institutional Life, Administration, National Insurance and Health.

No. 1435, Vol. LV. SATURDAY, DECEMBER 27, 1913. PRICE ONE PENNY

### THE CENTRE OF CHRISTMAS BLESSEDNESS.

It may be useful and helpful not only for the voluntary hospitals, but to the multitude of outsiders who take no part in their work, except perhaps to give occasionally to their support, to try and estimate the humane influence which the hospitals exercise upon everybody, from the physician to the patient, who is intimately associated with their work. Any governor of a hospital, and, indeed, every man or woman of wholesome feeling and heart, by setting aside an hour or two on Christmas Day to visit the hospital in which they may be interested, or to the work of which they have felt some attraction, may obtain inspiriting and helpful knowledge of this humane influence exercised by the hospital. No such visitor can fail to be struck with the loving care for the patients, and especially for the children, which is everywhere forthcoming. These characteristics are not confined to one ward but are common to all. In the case of hospitals which have the advantage of being attached to a medical school there will be found the most brilliant decorations, accompanied by every effort which the energy of youth and the best instincts of early manhood can produce, to cheer the patients, and make them happy and forgetful of their sufferings. The outside world little realises the excellence and beauties which the combined efforts of students, sisters, nurses, with the medical and lay staffs introduce into our voluntary hospitals at Christmas-time. Our message to our readers this Christmas is: Go to some hospital on Christmas Day, enter into the spirit and the goodness of all those, young and old, who have spontaneously given up the entire day to the attempt to bring Christmas peace and joy to the hearts of the suffering and the sick. In this way they will benefit themselves by realising the blessedness to be derived from an appreciation of the actualities and loving care lavished upon the sick. They will, further, undoubtedly gain a stimulus thereby which must tend to make them happier men and women than they have ever been before.

It is the habit of most people to go to a theatre or some other place of amusement frequently. Pageants, mystery plays, and picture shows are becoming more popular every year. None of these things will give so much genuine joyousness to the really intelligent beholder, as a spontaneous visit to some hospital at this season, when it is freely open to all. We wish every worker in every palace of pain throughout the land a blessed Christmas and a most prosperous and happy New Year. In our Christmas Appeal Number we publish some striking accounts by the secretary, the matron, the sister, and the nurse of the amount of work which falls to the lot of hospital residents at Christmas-time, and of the enjoyment which accrues to the workers, who enter upon these extra duties in the right spirit. It is an arduous period for hospital workers, and everyone who is associated with it gains inspiration and gets closer to the individual patient. So the whole moral influence of Christmas in the hospitals of this country is fruitful in beneficial results to all concerned. We are very anxious indeed, however, that the personal service rendered by the resident staff of all grades in our hospitals should be the limit to the call upon them at this season.

Of course, the Christmas festivities, the decorations, and entertainments in our hospitals cost money. That money ought to be provided from outside sources, and not one penny of it should come out of the pockets of the resident and working staff. We venture to hope that the practice now generally adopted in the best-administered hospitals of securing the co-operation, through the chairman or some active member of the committee, of a few outside governors to provide funds to defray these necessary charges, will, without exception, be adopted in every institution of the kind. It is not right that hospital workers should be permitted, however willing they may be, to pay at this season in meal and malt. Let some one person, outside the resident staff, connected with every voluntary hospital in the United Kingdom, make it his business to see that no worker has been overweighted by being called upon to pay the cost of making Christmas in the hospital what it ought to be, and what it undoubtedly has become in this country. As an old hospital resident of many years' standing, the remembrance of Christmas with the sick is one of the most precious possessions, which life has brought in its train. We extend the hand of comradeship and good cheer to our fellow-workers for the sick, everywhere, at this season. We pray that we and they may have good reason, through personal service, to remember with thankfulness the Christmas of 1913.

